# Herniotomy

**Published:** 1872-09

**Authors:** D. W. Hammond

**Affiliations:** Macon, Georgia


### HERNIOTOMY.

BY D. W. HAMMOND, M.D., MACON, GEORGIA.

[Read Before the Macon Medical Association, and Published by Order of said Body.]

Called to see James Mathews, a colored man, on the 18th June last, æt. twenty years—a drayman by trade. Had suffered from right inguinal hernia for two or three years; but by wearing a truss, it was easily controlled, and did not in the least incapacitate him for his daily yet laborious avocation.

The day previous to my visit to him, he had removed the truss for the purpose of bathing in the Ocmulgee river. Shortly after getting into the water, a large portion of intestine and omentum extruded through the abdominal ring, a considerable bulk of which descended into the scrotum, which he could not reduce. When I saw him, the scrotum was quite as large as a cocoa-nut—had a doughy feel, and projected over and above the inguinal canal. Failing to reduce it by the *taxis*, I ordered him placed in a warm bath, and administered a large dose of opium, to be repeated in three hours if the bowel did not return into the abdominal cavity.

On my second visit, six or eight hours subsequently, I found the patient in *statu quo*. Ice was applied freely around the scrotum and over the tumor for several hours, but without any alleviation. Discontinued the ice, gave a tea-spoonful of laudanum, and ordered the dose to be repeated in four hours. This was five o'clock on the evening of the 18th. At four o'clock A.M. of the 19th, stercoraceous vomitings commenced. Saw him next morning at nine o'clock, when I determined to operate at once, and requested the assistance of my friends, Drs. Nottingham, Holt and Green. He was placed on a table of convenient hight, and two feet wide. The bladder and rectum having been previously emptied, the parts of the seat of operation were shaved, and the patient placed under chloroform by Dr. Nottingham. An incision was made over the axis of the tumor, from two and a half to three inches in length—from above downward, by pinching up a fold of the integument, and transfixing it at its base with a sharp-pointed bistoury, and slitting it upward, making a linear incision the full length of the tumor. The adipose tissue and superficial fasciæ were dissected off and separated, and the sac carefully exposed by dividing consecutively the sub-serous laminated structures. The subject being young, and the rupture of short duration, there was no agglutination of the layers. The infundibular fascia, cremaster muscle, and the intercolumnar tunics, were severally divided, one by one, until the tendon of the external oblique muscle was brought into view. The sac had lost, to some extent, its shining appearance, and was dark and congested, yet had not lost its normal temperature.

We had now arrived at the most difficult stage of the operation—viz., the opening of the sac—in the accomplishment of which the intestine is frequently injured. Fold after fold was lifted up and cut, until a small opening was made into the cavity, from which issued two ounces or more of dark serous fluid, which had no "fœtid smell." By the grooved director and bistoury, it was slit up and down to the full extent of the original section. The knuckle immediately protruded, presenting a dark livid hue, and surrounded by a still darker sheet of impacted omentum—a case of entero-epiplocele of the books. On exposing the strangulated bowel, it was found that the peritoneal coat had been touched with the knife, or scratched by the tenaculum, making a wound about the fourth of an inch in extent, from which *accident* several drops of dark grumous blood oozed out.

The stricture, on the introduction of the finger, was found to be at the neck of the sac, which is generally the case in inguinal hernia; this I divided upward and outward by the herniotome, and attempted to return the bowel, but was foiled for want of sufficient space. Two or three more separate incisions on the outer and inner margins of the contracted tissues were made, which enabled the bowel readily to return. The dark omentum was completely injected, with clots of coagulated blood between the laminated folds. The entire mass was drawn down as far as possible, and cut off. There being no hemorrhage, it was sponged well and returned *without tying*. The quantity removed weighed over four ounces.

The wound was closed by four sutures and adhesive strips, and the ordinary bandages properly adjusted; a half grain of morphine administered, and the patient placed in bed. Had a good recovery from the chloroform; the pulse was feeble and steady. Gave whisky freely which caused reaction to take place in about one hour.

Saw him again at six o'clock P.M. Pulse one hundred and ten per minute, and full; resting quietly. Gave a tea-spoonful of laudanum, and ordered it to be repeated during the night, if necessary. Saw him next morning. Was informed that he rested well during the night, without repeating the anodyne.

20th.—Quiet; pulse somewhat excited; pain in the wound, and slight orchitis. Applied cold cloths over the bowels and scrotum, and gave forty drops tinct. opii.

21st.—Restless during the night; pulse ninety-five, with slight tympanitic distension. Ordered rice-water and turpentine emulsion—each table-spoonful of which to contain ten drops of the turpentine, to be repeated every four hours—with thirty drops tinct. opii occasionally.

22d.—Nine o'clock A.M., found him with less pain, and abdomen less distended. Removed the adhesive strips, and dressed the wound, and applied a compress saturated with a weak solution of carbolic acid, and ordered fifty drops laudanum.

23d.—Rested well. Continued same dressings; removed two of the sutures; wound discharging freely of dark, offensive fluid; ordered beef tea, and forty drops tinct. opii.

24th.—Passed the night comfortably; pulse eighty-six. Continued dressing and emulsion; the two remaining sutures were removed; dark, bloody fluid, intermixed with pus, discharging from the wound; repeated the anodyne, and directed the beef tea to be continued, with a little boiled rice in addition.

25th.—Feeling comparatively comfortable; some appetite; discharges diminishing, and assuming a more healthy appearance; wound adhering over the ring. Anodyne continued, together with the turpentine.

26th.—Doing well; continued treatment.

27th.—Improving; wound adhering throughout its whole extent; discharge slight; some wind passing from the bowels, with pressure in the lower part of the abdomen. Ordered an enema, which operated finely, and greatly to his relief—the first evacuation from his bowels since the operation.

28th to 30th.—Gradually recovering; wound still closing, with but little discharge; is able to sit up in bed, and has considerable appetite. Allowed him a soft boiled egg and toast.

By the 10th of July, the wound was firmly cicatrized, and he was able to walk about the house without difficulty. In six weeks, he was able to do slight work. I directed him to reapply the truss, which he did; but I was informed by his father that he had abandoned it, as he thought it was of no use. I have examined him recently, and find that the parts seem to be firmly condensed: and he may be, doubtless, radically cured.

Persons operated on for hernia frequently recover under the most unfavorable circumstances. The bowel may be completely engorged and its textures softened, and may slough, and large portions of the omentum become disorganized from sphacelus, and yet the subject have a safe recovery. Sir A. Cooper mentions a case of strangulated hernia, in which both omentum and intestine had been returned; and a short time after, the patient complained of such intense pain that the ligatures were clipped and the wound opened, when a large gangrenous fold of the omentum protruded, and continued to descend until several ounces sloughed away, and the patient recovered. Another case, receutly reported under the care of Callaway, in a man forty-two years of age, survived after losing seven and a half ounces of gangrenous omentum.

In the case just reported, the prognosis, under all the circumstances, was unfavorable for recovery. But may not the success of the operation be attributable to the treatment? The bowels were confined for nine days after the operation, by large doses of opium. This plan of treatment I adopted by the earnest solicitations of the physicians who assisted me in the operation. And may not this course of treatment have prevented peritoneal inflammation? It is true, however, that cold applications and turpentine emulsions were used in conjunction. I am of the opinion that many cases of this kind could be cured *without dilating the stricture or enlarging the inguinal canal.* When a large fold of the bowel is suddenly forced out, and at the same time distended with flatus, thereby rendering it irreducible from distension, why not let out the gas at once by pricking the bowel with a grooved needle?—or better, by a hollow needle, such as found in a hyyodermic syringe, which, I think, would be an admirable instrument for this purpose. All that would be required would simply be to unscrew the top of the syringe, and then pierce the bowel in an oblique direction, and across the muscular fibres, so that the needle would make a valvular opening. As the air passed out through the tube, the bowel would be collapsed by contracting upon itself. This would effectually close the opening made by the hypodermic needle. Such a procedure strikes my mind with such force that, should I ever have another case of the kind, I should most unhesitatingly give it a trial; and I most respectfully recommend it to the consideration of the profession. Flatus has been frequently evacuated from a strangulated bowel by the grooved needle. It has been practiced repeatedly at Guy's Hospital, and with success. Stocker, who is said to have more clinical experience than any surgeon in London, advocates its use. He has used it in ileus, "and with immense relief." "The cow-keeper taps his cows, and they recover."

Thomas Bryant, Esq., relates a case in an old man, aged seventy-one years, who was operated on for strangulated hernia; the wound closed, and was properly brought together by means of a pad and spica bandage of strapping. During the night, however, he got out of bed, and tore off the dressings, and kicked up a general hubbub about the house. As a consequence, the bowel protruded again, larger than ever. Every effort was made to reduce it, but without success. As a last resort (rather than let the old man *die*), the bowel was *punctured in four or five places* with a grooved needle, the wind passed out, and the bowel quietly passed back; the wound was readjusted and firmly secured. The Doctor observes: "I am pleased to add, no one bad symptom followed these rough measures, and a good recovery ensued."

The favorable result in the above case should cause the intelligent physician to pause and reflect. The practitioner has a great deal yet to learn—this is a progressive age. And I will here venture the opinion, that if it could so happen that one hundred patients with strangulated hernia had to be operated on, and fifty of them in the usual manner, and fifty by the hypodermic needle, or by a similar instrument made for the purpose, that the latter would be twenty-five per cent. more successful. In the usual operation, chloroform is generally administered, which sometimes produces death. It also requires a large wound in a dangerous location. The peritoneum has to be cut, and may inflame, which endangers the patient's life. The bowel is sometimes accidentally wounded; the spermatic cord and vessels are involved; and further still, the epigastric artery is frequently cut, and may produce a fatal hemorrhage. The hypodermic method, or the puncturing plan, can be done without chloroform or the knife. The tumor can be directly pierced by the needle, and the gas let out, and in a majority of cases, returned withont difficulty. Here there is no wound to stitch and nurse and dress for ten or fifteen days. There is no breech of continuity, comparatively speaking, and the patient is relieved at once. But, peradventure, it may be asked: Suppose this plan should fail? I reply: Then the patient, in the last resort, can avail himself of a more severe and dangerous operation, which may or may not save his life.

